# *OBV* (obscure vein), a C_2_H_2_ zinc finger transcription factor, positively regulates chloroplast development and bundle sheath extension formation in tomato (*Solanum lycopersicum*) leaf veins

**DOI:** 10.1038/s41438-021-00659-z

**Published:** 2021-11-01

**Authors:** Jinghua Lu, Chunyang Pan, Xin Li, Zejun Huang, Jinshuai Shu, Xiaoxuan Wang, Xiaoxiao Lu, Feng Pan, Junling Hu, Hui Zhang, Wenyue Su, Min Zhang, Yongchen Du, Lei Liu, Yanmei Guo, Junming Li

**Affiliations:** Key Laboratory of Biology and Genetic Improvement of Horticultural Crops of Ministry of Agriculture, Institute of Vegetables and Flowers, Chinese Academy of Agricultural Sciences, Beijing, 100081 China

**Keywords:** Plant breeding, Genetic mapping

## Abstract

Leaf veins play an important role in plant growth and development, and the bundle sheath (BS) is believed to greatly improve the photosynthetic efficiency of C_4_ plants. The *OBV* mutation in tomato (*Solanum lycopersicum*) results in dark veins and has been used widely in processing tomato varieties. However, physiological performance has difficulty explaining fitness in production. In this study, we confirmed that this mutation was caused by both the increased chlorophyll content and the absence of bundle sheath extension (BSE) in the veins. Using genome-wide association analysis and map-based cloning, we revealed that *OBV* encoded a C_2_H_2_L domain class transcription factor. It was localized in the nucleus and presented cell type-specific gene expression in the leaf veins. Furthermore, we verified the gene function by generating CRISPR/Cas9 knockout and overexpression mutants of the tomato gene. RNA sequencing analysis revealed that *OBV* was involved in regulating chloroplast development and photosynthesis, which greatly supported the change in chlorophyll content by mutation. Taken together, these findings demonstrated that *OBV* affected the growth and development of tomato by regulating chloroplast development in leaf veins. This study also provides a solid foundation to further decipher the mechanism of BSEs and to understand the evolution of photosynthesis in land plants.

## Introduction

As the vascular system in leaves, leaf veins play a critical role in transporting water and nutrients to leaves, thus supporting the development of higher plants^1^. In general, leaf veins vary greatly in arrangement, size, density, and geometry of the phloem and xylem vessels depending on the species. Vein structure and patterning are tightly related to their functions and ecological distribution^2^. For example, thicker veins are beneficial to rice plants under water-deficit conditions^2,3^. In leaf veins, bundle sheath cells (BSCs) form a wreath-like structure around the vasculature and connect the vascular bundle to the epidermis. The most important role for BSCs is to partition photosynthesis between the bundle sheath (BS) and mesophyll cells in C_4_ plants, improving the efficient fixation of CO_2_, particularly under warm and dry conditions^4–7^. Although the BS of C_3_ plants is less important for photosynthesis than that of C_4_ species, it has a vital role in the control of hydraulic conductance^8^, the transport of metabolites in and out of the veins^9^, responses to high light episodes^10^ and the assimilation of sulfur^11^. In addition, the presence or absence of bundle sheath extensions (BSEs) during leaf development, referred to as heterobaric or homobaric, respectively, is a clear and typical trait that is closely linked to the ecological distribution^12^. BSEs have multiple functions, including leaf support, herbivore deterrence, gas diffusion, and hydraulic conductance^13–16^.

Although much effort has been made to understand BS and BSEs, most have focused on physiology and biochemistry^15,17–19^. Information is lacking from the molecular perspective. To date, several genes have been shown to control leaf vein development. For example, *DL*, a member of the *YABBY* gene family, regulates midrib formation^20^. *COV1*, a Golgi transmembrane protein, negatively regulates vein differentiation^21,22^. The *PIN* gene family results in the proliferation of vascular tissue^23^. *DOF* genes, a class of plant-specific transcription factors, seem to be tightly associated with regions of vascular strand formation at the early stages of vascular development^24^. *LMI1-like*, an *HD-ZIP I* transcription factor, and *KNOX1*, a class I *KNOTTED1*-like homeobox transcription factor, coordinately control leaf vein patterns^25^. The phloem-expressed KANADI (*KAN*) family of transcription factors represses procambial formation through *PIN* protein-mediated auxin transport^26^. *Auxin response factor* genes independently or overlappingly function in vein development^27^. *OsNUS1*^28^ and *OsARVL4*^29^ affect vein color to different degrees by regulating chlorophyll content and chloroplast development. In addition, evidence from cell type-specific gene expression in BS has demonstrated that a special region, such as a *cis*-element, is necessary to drive cell type-preferential expression^6,7,11,30–34^. The role of the BS, however, is still less clearly defined, particularly in C_3_ plants^6^. Hence, the detailed regulatory mechanisms of the development and formation of BS or BSE need to be addressed.

As an excellent model plant for berry fruits, tomato is cultivated worldwide, and substantial amounts of data on this dicotyledonous C_3_ crop have been documented. In terms of the leaf vein, all wild species and most cultivars possess clear transparent veins, with the exception of the *obv* mutant, which presents dark veins^35^. The *obv* allele was continually selected by breeders for incorporation into the processing of tomato varieties for mechanical harvesting^36^. It was found that this allele was associated with gains in leaf gas exchange-related traits^37^. Previously, it was suggested that *obv* might be responsible for the chlorophyll content in the leaf vein^36^, which was later confirmed based on the absence of BSEs in the *obv* mutants^18^. Several investigations have been conducted on physiological traits to understand the functions of this allele but have been limited to analyzing its effect on carbon economy and growth^18,19^. Alternatively, genomic information might aid in deciphering this puzzle. Moreover, the *obv* mutation might be a very good material to uncover the formation, differentiation, and development of leaf veins in C_3_ plants. This study may provide additional information on the evolution of photosynthesis in land plants.

The *OBV* gene has been mapped on tomato chromosome 5 with a region of approximately 1.5 cM^36^. In this study, we cloned *OBV* by combining map-based cloning and genome-wide association study (GWAS) approaches and further verified the function of the gene by CRISPR/Cas9 and overexpression technology. The differentially expressed genes (DEGs) in the mesophyll and BSCs were also investigated using RNA-seq. The results provide additional evidence to elucidate the formation and regulatory mechanisms of the leaf vein.

## Results

### Characterization of *obv* and physiological traits

**Fig. 1:**
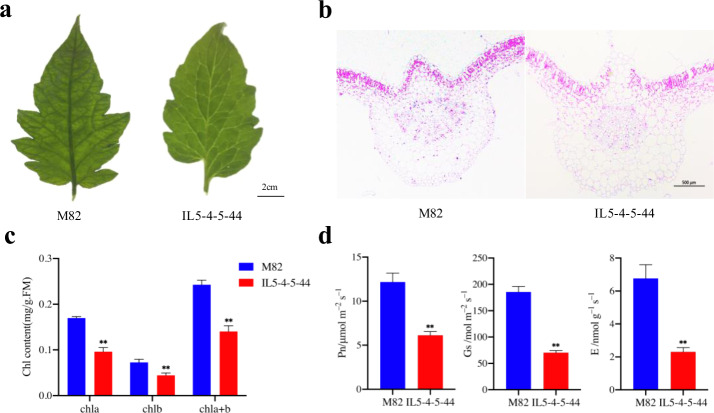
Phenotypic observations of IL-5-4-5-44 and M82. **a** Phenotypes of the fourth true leaf in M82 and IL-5-4-5-44 plants. **b** Cross sections of M82 and IL-5-4-5-44 leaf veins. **c** Chlorophyll content in the leaf veins of M82 and IL-5-4-5-44. **d** Photosynthetic parameters. Asterisks indicate statistically significant differences determined using a *t*-test (*P < 0.05; **P < 0.01)

IL5-4-5-44 is a very short introgression fragment derived from IL5-4 in the genetic background of M82^38^. Previously, both IL5-4 (*OBV*) and M82 (*obv*) were used to map the *obv* gene^36^. Hence, we employed the near-isogenic lines (NILs) IL5-4-5-44 and M82 to investigate the phenotype of the *obv* gene and several physiological traits. We observed that the cotyledons already showed different veins between the two genotypes when the seedlings sprouted from the soil. As true leaf development progressed, it became easier to distinguish them from each other. In the fully expanded leaves, IL5-4-5-44 showed a transparent leaf vein, whereas M82 displayed an obvious dark vein phenotype (Fig. 1a). To investigate the BSEs, cross-sections of major veins were observed. In IL5-4-5-44, the BSCs extended to the epidermis and formed BSEs, whereas no extension occurred in M82 (Fig. 1b). We also measured the chlorophyll content of the major vein, and the results indicated that both the Chl *a* and Chl *b* in the veins of IL5-4-5-44 decreased by ~41 and 44% compared with M82, respectively (Fig. 1c). Consistent with the chlorophyll content, the photosynthetic assimilation rate, stomatal conductance, and transpiration rate decreased by approximately 50%, 62%, and 66% in IL5-4-5-44 when compared with M82, respectively (Fig. 1d). These results are consistent with those of Barrios-Masias et al^37^.

Considering the significant difference in photosynthetic parameters, we continued to observe the stomata in the vein and the lower epidermises. No stomata were detected in the vein for either genotype, whereas high-density stomata were observed in the lower epidermis (Supplementary Fig. S1a). Moreover, 65.3% and 24.5% of the stomata were opened in M82 and IL5-4-5-44, respectively, which was statistically significant (Supplementary Fig. S1b). Additionally, we found that the vascular bundles of both genotypes contained well-developed chloroplasts (Supplementary Fig. S1c), and the number of chloroplasts per 1000 μm^2^ cell area in IL5-4-5-44 was one-twentieth of that in M82, which was also statistically significant (Supplementary Fig. S1d).

### Cloning of *OBV* by combining GWAS and map-based methods

**Fig. 2:**
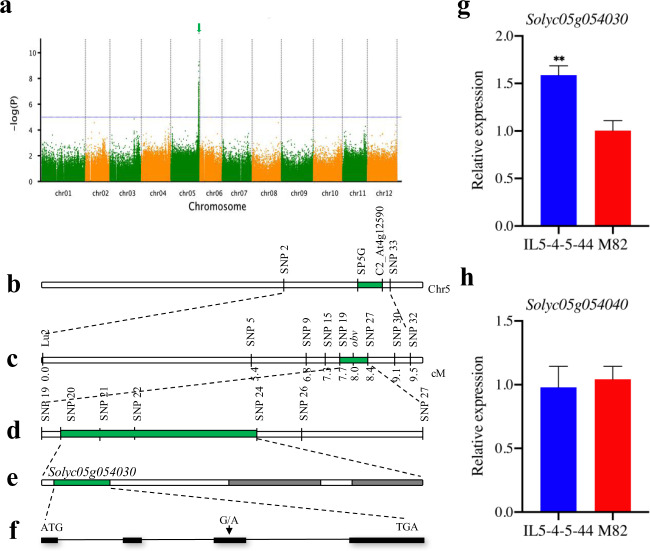
Fine mapping of *obv*. **a** Manhattan plot of GWAS for the obscure leaf vein. **b**–**f** Map-based cloning of the *obv* locus. Green arrow represents the most significant SNP association loci. **b** Green boxes represent the previous results of the fine positioning of *obv*. **c** Coarse linkage map of the *obv* locus on chr5. **d** The *obv* locus was narrowed down using 58 recombinants. **e** Three putative genes predicted in ITAG2.40. **f** *OBV* structure and the mutation site. Black boxes represent the coding sequences, and lines between boxes represent introns. **g**–**h** Relative expression levels of two genes in the leaf veins of IL-5-4-5-44 and M82 plants. A tomato ACTIN (*Solyc03g078400*) gene was used as the reference gene. Data are the mean ± SD of three biological replicates. Asterisks indicate statistically significant differences determined using a *t*-test (*P < 0.05; **P < 0.01)

The GWAS was conducted in a diverse population of 299 inbred lines. The fully expanded fresh leaves for each genotype were scored as described earlier. Among the 299 materials, 129 lines were clear-veined, 163 lines were dark-veined, and seven lines were scored as missing data (Supplementary Table S1). The whole-genome SNPs were screened based on the published tomato reference genome SL2.50. Ultimately, we obtained 125 high-quality SNPs (LOD > 5), and all of them were positioned on chromosome 5. The confidence interval region obtained by GWAS was chr05: 63,049,462 bp–64,012,700 bp, and the SNP with the highest LOD value was SL2.50 chr05: 63,989,747_G_A (12.91). The GWAS analysis demonstrated that the *obv* gene was located at the distal end of the long arm of chromosome 5 (Fig. 2a). These findings are basically consistent with a previously reported region spanned by markers SP5G and C2_At4g12590^36^ (Fig. 2b).

In view of the low recombination frequency between M82 and IL5-4^36,38^, we established a new F_2_ population by crossing 05-49 and 05-62 instead of M82 and IL5-4-5-44. A total of 37 SNP markers on chromosome 5 were obtained and used to screen the parental lines. Among the 37 SNP markers, 17 were polymorphic between the parents. We used eight markers to construct a genetic map with 1500 F_2_ individuals. Eventually, the *obv* locus was mapped to an interval between markers SNP19 and SNP27 on chromosome 5 (Fig. 2c). Based on the genotypes and phenotypes of 58 F_2_ recombinants, the *obv* locus was further narrowed down to a genomic region of ~24 kb between markers SNP20 and SNP24 (Fig. 2d; Supplementary Tables S2 and S7). According to the Solanaceae Genomics Network website (http://solgenomics.net), three candidate genes within the 24.14-kb genomic region were taken into consideration (Fig. 2e). Functional annotations pertaining to these candidate genes are shown in Supplementary Table S3. These three open reading frames (ORFs) included a transcription factor (*Solyc05g054030*), a transmembrane protein gene (*Solyc05g054040*), and a glutamic acid decarboxylase (*Solyc05g054050*).

To further detect the difference in the three candidate genes between the parental lines, we amplified the full length of the three candidate genes and sequenced them by Sanger sequencing. We found that there was no difference in the *Solyc05g054040* coding region, but we found one SNP (G–A) in the third exon region of *Solyc05g054030* and another SNP (T–C) in the seventh exon region of *Solyc05g054050*. However, the mutation in *Solyc05g054050* occurred far from the flanking marker SNP24. To verify these candidates, we performed qRT-PCR on both the parental lines and NILs (M82 and IL5-4-5-44) (Fig. 2g, h). All the primers used are listed in Supplementary Table S7. Compared with M82, only the expression of *Solyc05g054030* was significantly induced in IL5-4-5-44. A slight difference was observed for *Solyc05g054040* and no expression for *Solyc05g054050*. Hence, by combining the sequencing and qRT-PCR results, we predicted that ORF1 (*Solyc05g054030*) should be the candidate gene, which belongs to the C_2_H_2_ zinc finger structural transcription factor and contains four exons, a 1149-bp CDS region, and 381 translated amino acids (Fig. 2f). A single G-to-A substitution occurred in one ORF, *Solyc05g054030*, resulting in the substitution of amino acid Arg to His in the CDS.

### Sequence alignment and phylogenetic analysis

Amino acid sequence alignment of *OBV*-related protein sequences from Solanaceae plants and other selected species (*Nicotiana tabacum*, *Oryza sativa*, *Arabidopsis thaliana*, *Glycine max*, and *Vitis vinifera*) was conducted using MEGA 7.0 software. A BLAST search (http://blast.ncbi.nlm. nih.gov/Blast.cgi) revealed that only one copy of *OBV* was found in tomato species, and the OBV protein shared the highest sequence similarity with *Solanum pennellii* (98.96%), *Solanum chilense* (98.95%), *Capsicum chinense* (95.03%), and *Solanum tuberosum* (94.1%), and they all clustered within the same clade (Supplementary Fig. S2). However, *O. sativa*, *A. thaliana*, *G. max*, and *V. vinifera* clustered within another clade (Supplementary Fig. S2a). Amino acid sequence alignment showed that the ZnF-C_2_H_2_ domains were highly conserved among these proteins. In addition to the three predicted zinc finger motifs, these genes contained a highly conserved domain at their N-terminal region (Supplementary Fig. S2b). An amino acid substitution was found in the highly conserved domain in the *obv* mutant, suggesting that the conserved domain was important for the molecular function of *OBV*.

### Expression patterns of *OBV*

**Fig. 3:**
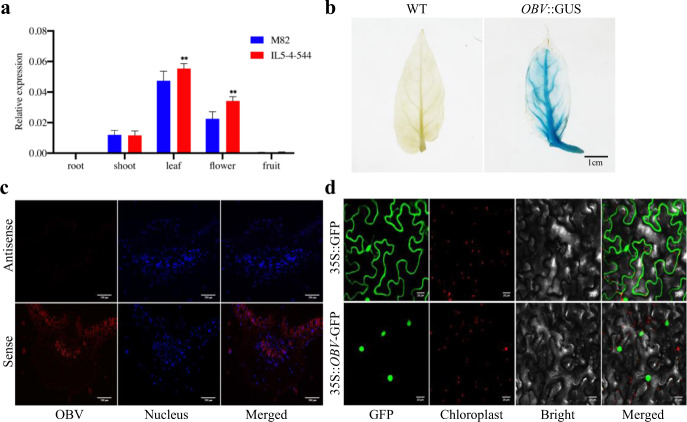
Gene expression profiles of *OBV*. **a** Quantitative RT-PCR analysis of *OBV* expression patterns in various tomato tissues of IL-5-4-5-44 and M82 plants. **b** Analysis of *OBV* promoter-driven GUS expression in young leaves. **c** FISH analysis of *OBV* expression in the leaf veins of Micro-Tom. A sense probe was used as negative control. **d** Subcellular localization of the *OBV*-GFP fusion protein. Scale bars: **b** 1 cm, **c** 200 μm, **d** 20 μm. Asterisks indicate statistically significant differences determined using a *t*-test (*P < 0.05; **P < 0.01)

Tissue type-specific gene expression is critical for plants to carry out diverse and specialized functions in distinct tissues. Thus, we assessed the expression of *OBV* in different organs. The results showed that *OBV* was mainly expressed in the leaves and floral organs, with a low signal in the shoots and a very weak signal in the roots and fruits (Fig. 3a). In addition, we constructed an *OBV*::GUS vector and transferred it to WT plants. GUS staining activity was mainly detected in the leaf veins (Fig. 3b). However, evidence from fluorescence in situ hybridization (FISH) further showed that the red fluorescence signal was present in the vascular bundles and palisade tissues of the cross-section of the leaf veins (Fig. 3c). As the OBV protein was predicted to be a nuclear protein on the UniProt website (https://www.UniProt.org/UniProt/A0A3Q7GPS4), we continued to seek more direct evidence using subcellular localization analysis. Transient expression of the *OBV*::GFP fusion protein in tobacco epidermal cells confirmed that the OBV protein was exclusively localized in the nucleus instead of the chloroplasts (Fig. 3d), which is consistent with its putative role in transcriptional regulation.

### Characterization of transgenic plants

**Fig. 4:**
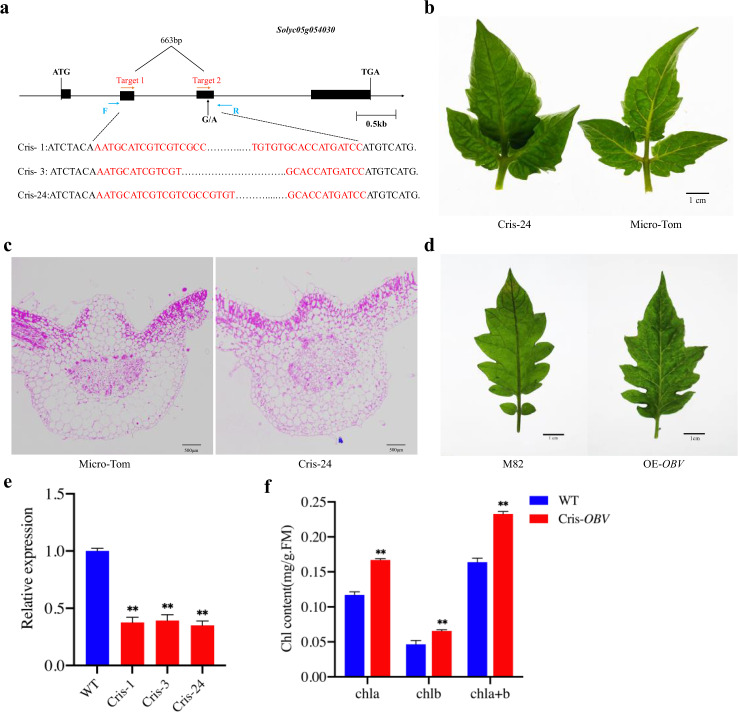
Phenotypic characterization of Cris-*OBV*, overexpressing OE-*OBV*, and wild-type (WT) plants. **a** Three deletion types of knockout mutants. **b** Phenotypes of leaf vein in Cris-*OBV*(Cris-24) and WT. **c** Cross sections of Cris-*OBV* and WT leaf veins. **d** Phenotype characterization of OE-*OBV* and WT (M82). **e** Expression levels of *OBV* in Cris-*OBV* leaf veins compared with that of WT. **f** Chlorophyll content. Values are the mean ± SD of three biological replicates. Asterisks indicate statistically significant differences determined using a *t*-test (*P < 0.05; **P < 0.01)

To verify the function of the putative *OBV* (*Solyc05g054030*) in tomato leaf veins, transgenic tomato plants with repressed *OBV* by CRISPR/Cas9 and overexpressed *OBV* by the 35S promoter were produced. We identified three independent *obv* mutants (Cris-1, Cris-3, Cris-24) in the Micro-Tom background (Fig. 4a) and overexpression plants in the background of M82 by PCR and sequencing (Fig. 4d). As expected, we observed significant differences in leaf veins not only between the Cris-*OBV* and WT (Micro-Tom) plants but also between the overexpression *OBV* (OE-*OBV*) and WT (M82) plants. The Cris-*OBV* plants showed obscure leaf veins (Fig. 4b), whereas the OE-*OBV* plants exhibited a change from dark veins into a transparent phenotype (Fig. 4d). The results of the paraffin sectioning experiments showed that the palisade tissue in the leaf veins of Cris-*OBV* was continuously arranged in the upper epidermis, whereas WT Micro-Tom presented a discontinuous pattern (Fig. 4c). This is completely consistent with results previously shown by NILs. Compared with WT, *OBV* expression was significantly decreased in the veins of Cris-*OBV* (Fig. 4e), which verified the effective repression of *OBV* in these transgenic plants. Taken together, these results led us to conclude that *Solyc05g054030* was the causal gene of *OBV* in leaf veins. Considering the significant difference in chlorophyll content presented in NILs, the chlorophyll content was also measured in WT and Cris-*OBV* leaves. In the Cris-*OBV* leaf, the content of both Chl *a* and Chl *b* was ~1.4-fold higher than that in WT (Fig. 4f). In addition, we observed that the leaf shape of OE-*OBV* changed and showed slightly small and slender leaves (Fig. 4d), which indicated that overexpression of *OBV* might regulate the hormone response to leaf development.

**Fig. 5:**
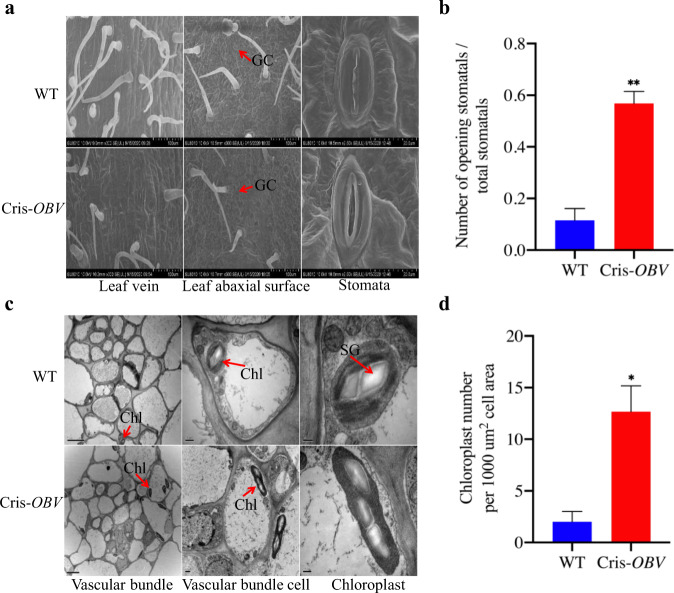
Electron microscope observations of *obv* and WT. **a** SEM observations of Cris-*OBV* and WT. **b** Analysis of the ratio of open stomata to total stomatal number in the lower epidermal cells. **c** TEM observations of Cris-*OBV* and WT. **d** Analysis of chloroplast number in the vascular bundle. GC, guard cell; Chl, chloroplast; SG, starch granule. Asterisks indicate statistically significant differences determined using a *t*-test (*P < 0.05; **P < 0.01). Scale bars: (a1–a4) 100 μm, (a5–a6) 200 μm, (c1–c2) 5 μm, (c3–c4) 0.5μm, (c5–c6) 0.2 μm

In addition, we also observed the chloroplasts and stomata of the Cris-*OBV* and WT leaf veins. Previous research has shown that stomata are frequently absent in the epidermis overlying vein paths because of the tight packing of parenchymal cells extending to the epidermis from the vascular BS^12,37^. Consistent with this view, there were almost no stomata on the leaf veins of the WT, whereas a few stomata were present on the veins of the Cris-*OBV* mutants (Fig. 5a). Statistical analysis showed that the stomatal opening rate of *OBV* was significantly higher than that of WT (Fig. 5b). Moreover, the number of chloroplasts was also significantly increased in Cris-*OBV* (Fig. 5c, d). Correspondingly, these results for OE-*OBV* and M82 (Supplementary Fig. S3a–f) were consistent with the performance of Cris-*OBV* and Micro-Tom.

### *OBV* regulates the transcription of genes associated with chlorophyll biosynthesis and photosynthesis

**Fig. 6:**
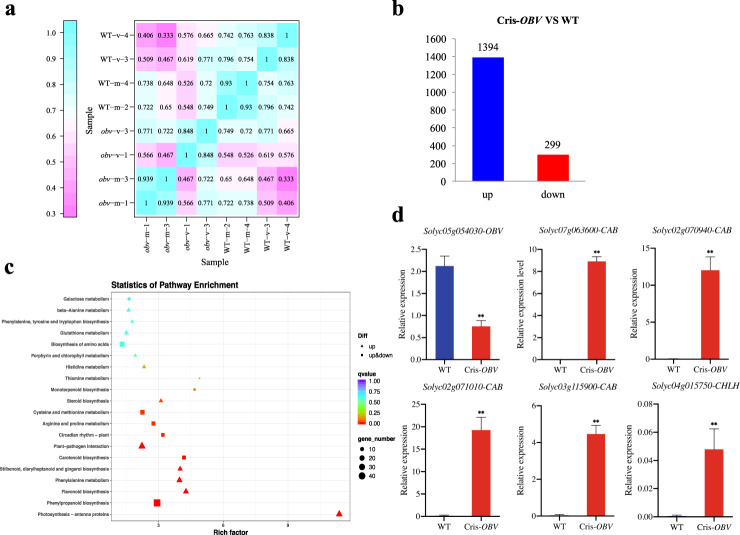
RNA sequencing analysis between Cris-*OBV* and WT. **a** Correlation statistics between the RNA-seq samples. **b** The number of genes that were upregulated and downregulated in veins of Cris-*OBV* compared with that of WT. **c** KEGG enrichment results of the DEGs. The number of genes is indicated by the size of the dots. The *P*-value of the metabolic pathways is indicated by the color of the dots. **d** The RNA-seq results were validated by qRT-PCR. The data represent the mean ± SD of three biological replicates. Asterisks indicate statistically significant differences determined using a *t*-test (*P < 0.05; **P < 0.01)

To further evaluate the regulatory mechanism of *OBV* in vein development, we performed RNA-seq of WT and Cris-*OBV* mesophyll and vein samples. The FPKM values of the two biological replicates for each sample were highly correlated, indicating that the RNA-seq data were reliable (Fig. 6a). For WT, 1027 differentially expressed genes (DEGs) were found (Supplementary Table S4). Corresponding to the mesophyll, 677 were upregulated and 350 were downregulated in the vein (Supplementary Fig. S4a). The Kyoto Encyclopedia of Genes and Genomes (KEGG) analysis (Supplementary Table S4) showed that the differences produced by the two distinct tissues were mainly associated with the plant hormone signal transduction and diterpene biosynthesis pathways, including 34 DEGs responsible for cell recycle control, cell division, auxin response protein, ethylene response factor, abscisic acid metabolism, jasmonic acid-amido synthesis, and gibberellin synthesis (Supplementary Fig. S4b). In contrast, *OBV* knockout caused 2607 DEGs (1648 downregulated and 959 upregulated) (Supplementary Fig. S4c and Table S5). The significantly enriched pathways were associated with photosynthesis-antenna proteins, photosynthesis, carbon fixation, carbon metabolism, and the pentose phosphate pathway (Supplementary Fig. S4c and S4d). Many genes in the photosynthesis-antenna proteins and photosynthesis pathways were downregulated and related to the light-harvesting chlorophyll protein complex (LHC) or photosystem I/II reaction center. We also found that the expression of genes related to chlorophyll and carotenoid metabolism was also downregulated (Supplementary Table S5).

We identified 1793 DEGs in the veins of the Cris*-OBV* mutant and WT, including 1394 upregulated and 299 downregulated genes (Fig. 6b; Supplementary Table S6). The KEGG analysis showed that *obv* affected multiple metabolic pathways, including photosynthesis-antenna proteins, phenylpropanoid biosynthesis, and flavonoid biosynthesis (Fig. 6c). Notably, 23 DEGs in the photosynthesis-antenna protein pathway were significantly upregulated, all of which encode chlorophyll a-b binding proteins and are related to photosynthesis. However, not all *CAB* genes in this pathway had the same upregulation multiple: Lhcb1 increased approximately 1206-fold, while Lhca2 only increased approximately 6-fold (Supplementary Table S6). In addition, the porphyrin and chlorophyll metabolism pathways were represented, which contain many genes involved in chlorophyll biosynthesis, such as *SlBEL11* (*Solyc11g068950*), *CHLH* (*Solyc04g015750*), and *CAOs* (*Solyc11g012850* and *Solyc06g060310*) (Supplementary Table S6). To test the reliability of RNA-seq data, we examined the transcript levels of six genes (*OBV*, *CAB*s, and *CHLH*) by qRT-PCR, and the results were consistent with the transcriptome data (Fig. 6d). Taken together, RNA-seq profiling suggested that the regulation of leaf vein development by *OBV* might be mediated by the transcription of genes associated with chlorophyll biosynthesis and photosynthesis in tomato.

## Discussion

### The *OBV* gene may cause changes in both the chlorophyll content and BSEs in tomato leaf veins

Tomato plants have compound leaves and show considerable shape diversity^39^. However, obvious variations in the vein, as the major component of the leaf, are so rare that one natural mutation named *obv* was first observed only in 1990^36^. This mutant presented dark veins compared with wild species and most cultivars and became more widespread in industrial tomato varieties for mechanical harvesting with the introduction of determinate growth in the 1930s^36^. Several studies have confirmed that the *obv* allele is associated with gains in leaf gas exchange-related traits and, together with another gene, *SP5G*, contributes to higher C assimilation and yield^37^. However, the cause of the dark character has not yet been completely determined. Direct comparison of transparent and dark veins suggested differences in the chlorophyll content of leaf veins^36,37^, while leaf anatomical observation indicated that the *obv* mutation eliminates BSEs in veins^18^. In this work, we confirmed the phenotype by combining two NILs and Cris-*OBV*. We found that well-developed chloroplasts were visible in the ultrastructure samples, and both M82 (*obv*) and Cris-*OBV* showed significantly increased numbers of chloroplasts in the major veins (Supplementary Fig. S1 and S3; Fig. 5d), which was consistent with the increased chlorophyll content by at least 1.5-fold in both genotypes (Figs 1c and 4f). This finding corroborates previous results whereby Det-Obs ILs still showed a slightly higher chlorophyll content than SemiDet-Clr even though the entire leaves were used for content measurement instead of the major veins^37^. Our transcriptome data again strongly supported these results, as 29 genes related to chloroplast development and chlorophyll synthesis were significantly increased in Cris-*OBV* mutants, such as *SlBEL11*, *CHLH*, and *CAOs* in the porphyrin and chlorophyll metabolism pathway and the CAB family in the photosynthesis-antenna protein pathway (Fig. 6c; Supplementary Table S6), which have been shown to play an important role in chlorophyll synthesis and photosynthesis^40–44^. However, BSEs were also clearly observed in both IL5-4-5-44 and Micro-Tom with transparent veins, as demonstrated by Zsögön et al.^18^. Thus, based on this accumulated evidence, we propose that the phenotype of the *obv* mutant should result from both the chlorophyll content and BSEs in tomato leaf veins instead of any factor alone.

### *OBV* is a C_2_H_2_ zinc finger protein that is specifically expressed in veins

To decipher the functions of the *obv* gene, we cloned this gene by combining GWAS and map-based cloning and completed functional verification by CRISPR/Cas9 and overexpression experiments. As expected, the CRISPR/Cas9 assay of *OBV* resulted in the dark vein phenotype in Micro-Tom (Fig. 4b), while its overexpression showed a transparent vein in M82 (Fig. 4d). Functional analysis indicated that *OBV* encoded a C_2_H_2_L domain class transcription factor with a typical C-X2-C-X12-H-X3-H single ZF structure located in the N-terminal region of *Solyc05g054030*. In tomato species, a total of 104 C_2_H_2_-ZFs have been identified^45^, most of which are associated with metal ion binding, DNA-binding transcription factor activity, the nucleus and regulation of transcription, and DNA templating. We found that *Solyc05g054030* was related to shoot gravitropism. Gravitropism is asymmetrical cell elongation between the upper and lower sides of organs^46^. A homologous gene of *OBV*, namely, *AtIDD14*, has been shown to cooperate with *IDD15* and *IDD16* to regulate lateral organ morphogenesis and gravitropism by promoting auxin biosynthesis and transport in *Arabidopsis*^47^. Another C_2_H_2_-type zinc finger protein, *SGR5*, is also involved in gravitropism in *Arabidopsis* inflorescence stems^48^. We hypothesize that *Solyc05g054030* may regulate the formation and morphology of veins through asymmetric cell elongation in tomato leaves. In plants, several genes, including *DL*^20^, *COV1*^21,22^, *PIN*^49^, *DOF*^24^, *LMI1-like* and *KNOX1*^25^, *ARF3*/*5*/*7*/*19*^27^, and *BSD2*^50^, have provided direct evidence for the differentiation, formation, and development of leaf veins. Specifically, the Rubisco chaperone BSD2 regulates chloroplast coverage in maize BSCs^50^ since *OBV* also causes changes in the chlorophyll content of leaf veins. These findings might contribute to our further understanding of the regulatory network of *OBV*. Phylogenetic analysis further showed that the homologous sequences from *O. sativa*, *A. thaliana*, *G. max*, and *V. vinifera* did not cluster together with the OBV protein, unlike those from Solanaceae crops (Fig. S2a). Hence, *Solyc05g054030* would differ from them and have unexplored functions.

Extensive studies have focused on cell type-specific gene expression of BS to reveal the evolution of C_4_ photosynthesis^7,50^. Our qRT-PCR results showed that *OBV* was expressed at the highest level in leaves, followed by flowers (Fig. 3a). GUS analysis further demonstrated that *OBV* was specifically expressed in leaf veins (Fig. 3b). Therefore, *obv* mutation might provide new clues to help explain cell type-specific expression in leaf veins, which might be related to the differences in photosynthesis between C_3_ and C_4_ plants. However, our FISH results further showed that *OBV* expression was located in the palisade tissue around the veins and vascular bundles instead of BSCs (Fig. 3c). One possibility is that *OBV* may regulate the expression of other genes in BSCs, such as growth hormone-related genes, which can cause the extension of BSCs^51^, just like SHR is essential for BSC fate specification but is expressed only in central vascular tissue^52^. Another feasible explanation is that *OBV* may only be expressed during the development or formation of BSCs. The expression information for *OBV* in BSCs may have been overlooked since we collected samples only from fully developed leaves.

In addition, we found that the leaf shape of OE-M82 was small and slender (Fig. 4d) and delayed the flowering time compared with that in WT. This result coincided with the qRT-PCR results whereby *OBV* was expressed at the highest level in leaves followed by the flowers. Auxin plays a critical role in the initiation, patterning, and morphogenesis of compound leaves in plants^39^. As indicated in previous work, auxin response factor-encoding genes also function in vein development^27^. In tomato, cytokinins also participate in compound leaf development^53^. Therefore, *OBV* might be involved in leaf vein formation and development by mediating the hormonal regulatory pathway.

### Gene expression patterns hint that the *OBV* gene alters photosynthesis

To understand the transcriptional network regulated by the *OBV* gene, we produced tissue-specific transcriptomes of leaf mesophyll and veins using fully expanded true leaves. Compared with leaf mesophyll cells, the transcriptome of the veins of WT was enriched in plant hormone signal transduction and gibberellin (GA) synthesis properties. In total, 34 DEGs were directly related to the regulation of the phytohormone response (auxin, ethylene, abscisic acid, jasmonic acid, and gibberellin), 25 of which exhibited higher expression levels (Supplementary Table S4). The formation of leaf vascular bundles and the functioning of water and nutrient transport are regulated by hormone-mediated pathways^23,54,55^. We suspected that the altered leaf shape of the OE-*OBV* plants might also be caused by hormones. However, the transcriptomes of the veins were completely different from WT in many aspects following the knockout of the *OBV* gene, particularly regarding photosynthesis-antenna proteins and photosynthesis and carbon fixation in photosynthetic organisms (Supplementary Fig. S4). This distinction also suggested that knockout of the *OBV* gene caused extensive variation in both the leaf mesophyll and veins, as indicated by the different physiological traits that occurred with the *obv* mutation^18^. However, the samples collected from the leaf mesophyll tissues were inevitably mixed with the minor veins. Isolation of bundle sheaths and mesophyll cells could provide more concrete evidence to address this issue^50^.

When the transcriptomes of the different veins were compared, we found that photosynthesis-antenna proteins were significantly enriched in the *obv* mutant and that the related 23 genes were completely upregulated (Fig. 6c; Supplementary Table S6). This finding is consistent with the increased chlorophyll content in the mid-veins of the Cris-*OBV* genotype (Fig. 4f). After considering the backgrounds, including leaf mesophyll and *OBV*, we found that both the enriched photosynthesis-antenna proteins and carotenoid biosynthesis might be related to the chlorophyll content. Hence, we concluded that the *OBV* gene might cause a change in photosynthesis in the leaf veins in tomato. Moreover, the phenylpropanoid biosynthesis pathway was also enriched (Supplementary Tables S4 and S6), which is involved in vascular development^56^. This pathway might be related to the formation of BSE. Considering *OBV* as a C_2_H_2_ zinc finger protein that is expressed outside of the bundle sheath, it is necessary to determine the DNA-binding sequence in the near future to elucidate the regulatory mechanism controlled by *OBV*, which would also provide support to explain the cause of the *obv* mutation and changes in various physiological traits.

## Materials and methods

### Plant materials and growth conditions

A total of 299 inbred tomato lines with a determinate growth type were released from our breeding program (Supplementary Table S1). Seeds were sown in the greenhouse, and seedlings for each genotype were transplanted into an open field and managed using normal field procedures. At least three leaflets from mature plants were sampled from one genotype and used for phenotyping of the vein. Obscure veins (*obv*) versus clear veins (*OBV*) were scored visually outdoors by holding a leaflet over a reflective surface. We used these collected data for the GWAS analysis. Among them, we used two inbred lines, including 05-49 with the *obv* gene as the female parent and 05-62 with the *OBV* gene as the male parent, to produce an F_1_ hybrid. One single F_1_ plant was self-crossed to develop an F_2_ population for fine mapping and candidate gene analysis. A total of 1500 F_2_ individuals were grown in the greenhouse at a temperature of ~25 °C/18 °C day/night. We scored the vein phenotype for each individual as described previously. Three accessions, *Solanum lycopersicum* Micro-Tom (*OBV*), M82 (*obv*), and IL5-4-5-44 (*OBV*), were kindly provided by the TGRC (Tomato Genetic Resource Center). IL5-4-5-44 is an introgression line derived from the wild species *Solanum pennellii* LA0716^38^. They were also grown in the greenhouse for further analysis.

### Paraffin sectioning

For histological analysis, midrib veins removed from fully expanded leaves were cut into 2 mm × 2 mm segments and fixed in 3.7% FAA solution (3.7% formaldehyde, 70% ethanol, 5% glycerin mixture) at 4 °C overnight. After dehydration and infiltration, the samples were embedded in paraffin and cut into 8-μm sections using a Leica RM2016 microtome (Leica Microsystems, Wetzlar, Germany). These paraffin sections were then dyed using the HE staining method and imaged under a Nikon microscope (Nikon DS-U3, Japan).

### Chlorophyll measurements

We measured the chlorophyll content of each leaf sample spectrophotometrically^57^. A fully expanded, well-exposed fourth true leaf of M82 and IL5-4-5-44 was cut off, and the primary and first secondary vein were carefully removed using a scalpel. The weighed samples (0.2 g) were immediately transferred to a 10-mL centrifuge tube containing 8 mL anhydrous ethanol to extract the chlorophyll, and they were stored in a refrigerator at 4 °C for 48 h. We performed all determinations in triplicate. Subsequently, the samples were centrifuged at 8000 r/min for 5 min at 4 °C. The supernatant of the chlorophyll solution was placed in a cuvette for quantification of the chlorophyll content at 665 nm and 649 nm with a Lambda 900 UV/VIS spectrophotometer (PerkinElmer Inc., Waltham, MA, USA). The entire process of leaf chlorophyll extraction was conducted in a darkroom to avoid chlorophyll decomposition. We calculated the contents of chlorophyll *a* (Chl *a*) and chlorophyll *b* (Chl *b*) in the leaves using the formulas modified by Lichtenthaler (Lichtenthaler, 1987). The content of Chl *a* (μg/mL) = (13.95OD_665_-6.88OD_649_) × V/1000 W; the content of Chl *b* (μg/mL) = (24.96OD_649_ − 7.32 OD_665_) × V/1000 W. The OD_665_ and OD_649_ values are the absorbances at wavelengths of 665 and 649 nm, respectively. V (mL) is the volume of each sample solution, and W (g) is the fresh weight of each sample.

### Scanning electron microscope (SEM) observation

To observe the possible variation in the stomata caused by *obv*, we collected mid-regions of distal leaflets from fully expanded fourth true leaves, which were then cut into 5-mm segments and fixed in 3.5% glutaraldehyde for ~24 h at room temperature. After washing in 0.1 M phosphate buffer (pH 7.4) for 10 min, these samples were fixed in 1% osmic acid for 2 h, dehydrated in a graded ethanol series (alcohol concentration from low to high: 30%, 50%, 70%, 80%, 90%, 95%, 100%), and dried in a Leica-EM CPD 300 desiccator (Leica, Frankfurt, Germany), after which they were coated with a film of gold using a Hitachi MC1000 (Hitachi, Tokyo, Japan). Finally, observations and photos were carried out on a Hitachi SU 8010 scanning election microscope (Hitachi, Tokyo, Japan).

### Transmission electron microscope observation

We excised the samples from the fourth true leaves of the M82 and IL5-4-5-44 seedlings. A fragment of the primary vein (approximately 1 × 2 mm) was immediately fixed in 2.5% glutaraldehyde. A mild vacuum (~20 mbar) was applied until the leaf pieces sank. After the samples were fixed overnight at 4 °C, they were washed three times with 0.1 M phosphate buffer for 30 min and then postfixed with 1% osmiophilic tetroxide for 2 h. These fixed samples were dehydrated with a series of alcohol solutions and then infiltrated and embedded in Spurr’s resin (SPI-812) with an acetone mixture. The ultrathin sections were cut with a diamond knife on a Reichert Ultracut-6 (Leica Microsystems, Bannockburn, IL, USA), stained with uranyl acetate and lead citrate, and finally viewed with a transmission electron microscope (Hitachi HT7500, Tokyo, Japan) operated at 80 kV. Micrographs were obtained using a Gatan 832 digital imaging system^58^.

### DNA extraction

We isolated DNA from the young leaves of 5- to 6-week-old seedlings using the cetyl-trimethylammonium bromide (CTAB) method and diluted the DNA to a concentration of 100–150 ng/μL in RNase (10 mg/mL) H_2_O (1:100). We further determined the concentration of each DNA sample using an ultraviolet spectrophotometer. Approximately 200 ng of genomic DNA was used as a template for subsequent polymerase chain reaction (PCR)-based genotyping.

### GWAS

We performed GWAS on the resequencing data (0.5×) from 299 inbred lines of tomatoes. The raw data were submitted to the SRA (Sequence Read Archive) (http://www.ncbi.nlm.nih.gov/sra/), and the accession number was PRJNA708163. The paired-end reads of these accessions were mapped to the tomato reference genome (SL2.50). Single nucleotide polymorphism (SNP) calling was performed on the alignment results using the Genome Analysis Toolkit (GATK) (McKenna et al., 2010). We used more than one million high-quality SNPs (minor allele frequency [MAF] >5% and missing rate <30%) to perform GWAS for the phenotype of the obscure and clear veins in 299 accessions. We performed the association analyses using a mixed linear model (MLM) in TASSEL 5.0 software^59^. The cutoff *P*-value was set as 1E ^− 5^, and the SNP association sites were visualized using a Manhattan graph in R software (www.r-project.org).

### Mapping and cloning of the *obv* gene

To locate candidate genes, all sequences within linkage disequilibrium (LD) decay centered on the significantly associated SNPs were extracted and developed into Kompetitive allele-specific PCR (KASP) markers. Genomic DNA was extracted from the young leaves of 1500 F_2_ individuals as described earlier. Polymorphic SNP markers between the parents and F_1_ were chosen to identify the genotypes of the F_2_ individuals. The genetic linkage map for the F_2_ population was constructed using Joinmap 4.0 software^60^. Subsequently, the target region was gradually narrowed using recombinants and additional molecular markers. After fine mapping, we predicted candidate genes and analyzed their functions in SGN (https://solgenomics.net/). The supposed candidate gene was further confirmed by Sanger sequencing, and the sequences were aligned to determine the mutation in the coding region sequence. The primers used for fine mapping are listed in Supplementary Table S7.

### RNA isolation and gene expression analysis

We validated the expression of the candidate genes using quantitative real-time PCR (qRT-PCR). Total RNA was extracted from fresh young leaves using an RNA Pure Kit (Aidlab, Beijing, China) and treated with DNase I (Thermo Scientific, Waltham, MA, USA). First-strand cDNA was synthesized using TransScript One-Step gDNA Removal and cDNA Synthesis SuperMix (Transgene, Beijing, China). The qRT-PCR assay was performed using SYBR Green reagent (Yeasen, Shanghai, China) and the LightCycler 480 Real-Time detection system (Roche, Basel, Switzerland). We designed the primers used for qRT-PCR using Premier 5.0 software with annealing temperatures of 60–63 °C and amplification lengths of 100–200 bp. For the qRT-PCR analyses, the tomato *Actin* (*Solyc03g078400*) gene was used as the internal control. All primers used for qRT-PCR are listed in Supplementary Table S7. PCR amplification included a 10 min denaturation at 95 °C, followed by 40 cycles of 95 °C for 10 s, 57 °C for 20 s, and 72 °C for 20 s. Each gene expression analysis had three independent biological and three technical repetitions. We calculated the relative expression levels using the 2^−ΔΔCt^ method^61^.

### Sequence alignment and phylogenetic analysis

The deduced amino acid sequences of the *OBV* gene were collected from the NCBI database (https://www.ncbi.nlm.nih.gov/). A total of 14 sequences of the C_2_H_2_L domain class transcription factors were aligned. We analyzed the amino acid sequences encoded by this gene and the position of variation to further confirm the candidate gene. A phylogenetic tree was constructed using the neighbor-joining (NJ) method in MEGA v 7.0^62,63^. Evolutionary distances among the proteins were calculated using the Poisson correction method. We calculated bootstrap values based on 1000 replicate trees, and the values are shown at the corresponding branch nodes.

### Subcellular localization analysis

To determine the subcellular localization of the OBV protein, the CDS fragment of *OBV* without the stop codon was amplified and inserted into the expression vector to produce the fusion construct pBWA(V)HS-*OBV*-OsGFP-3HA using a ClonExpress II One Step Cloning Kit (Vazyme, China). Then, pBWA(V)HS-*OBV*-OsGFP-3HA and the control vector were transferred to *Agrobacterium tumefaciens* strain GV3101 and injected into the epidermal cells of 4-week-old tobacco leaves^64,65^. After 48 h of infiltration, we analyzed the subcellular localization of *OBV*-GFP using excitation/emission filters for green fluorescent protein (GFP) fluorescence (Ex/Em, 488/510 nm) and Chl fluorescence (Ex/Em, 640/675 nm) with a laser confocal microscope (Nikon C2-ER, Nikon Microsystems, China).

### Fluorescence in situ hybridization

We performed FISH with paraffin wax. The leaf veins of Micro-Tom, which were approximately 1 cm in length, were fixed for 24 h at 4 °C in freshly prepared 4% (w/v) paraformaldehyde buffered with phosphate-buffered saline^66^ (PBS, pH 7.2). Fixed tissues were dehydrated in a graded ethanol series and impregnated with paraplast (P3683-1kg, Sigma). They were then cut into 10-μm slices using a microtome (Leica Microsystems, Wetzlar, Germany). To generate the antisense and sense probe, the specific CDS region (408 bp) was amplified using the Digoxigenin RNA labeling kit (Roche, Switzerland). The primers used to amplify the template are listed in Supplementary Table S7. Dewaxed slices were mixed with the two probes (2 ng/μL) and hybridized in hybridization buffer for 12 h at 55 °C. The sections were then transferred onto slides when the signal was sufficiently strong, and images were obtained using a fluorescence microscope (Nikon Eclipse Ci, Japan). The excitation/emission (Ex/Em) filters for blue and red fluorescence were 380/420 nm and 560/590 nm, respectively.

### Vector construction and plant transformation

We amplified the full-length CDS of the *OBV* gene from Micro-Tom (WT) cDNA and then cloned it into the pBI121 expression vector, in which gene expression was driven by the CaMV 35S promoter. *OBV* knockout mutants were generated in Micro-Tom using the CRISPR/Cas9 system. For subcellular localization analysis, the full-length CDS of *OBV* was cloned into the pBWA(V)HS-OsGFP vector to yield a fusion protein with GFP at the C-terminus. For the β-glucuronidase (GUS) assays, the fragments of the *OBV* promoter were amplified from the genomic DNA of Micro-Tom and fused to the pBI121 vector harboring the GUS reporter gene. The empty pBI121 was used as a negative control. All final vectors were transferred into GV3101 using an *Agrobacterium*-mediated transformation method^67,68^. We identified the positive transgenic plants by PCR and sequencing analysis. For the GUS assays, the collected samples were immediately incubated in 5-bromo-4-chloro-3-indolyl-β-D-glucuronic acid reaction medium overnight^69^ and then dehydrated in 95% ethanol. Photographs were obtained under a binocular microscope. All pertinent primers are listed in Supplementary Table S7.

### RNA-seq analysis

To identify transcripts involved in the regulation of clear and obscure veins in tomato, we performed an extensive transcriptomic analysis of the leaf veins at the third true leaf stage. Total RNA was extracted from the leaf veins of WT Micro-Tom and knockout mutants with the RNAprep Pure Plant Plus Kit (Tiangen, Beijing, China). Samples were collected with two biological replicates. cDNA libraries were then constructed, and 150-bp paired-end reads were sequenced using NovaSeq 6000 (Illumina, San Diego, CA, USA). Raw reads were obtained and then trimmed and filtered using Trimmomatic v0.33 software to remove adapters and low-quality bases. The raw data were submitted to the SRA (http://www.ncbi.nlm.nih.gov/bioproject/707317), and the corresponding accession number was PRJNA707317. The clean data were then mapped to the reference genome (version SL2.50). Subsequently, gene expression levels were estimated with fragments per kilobase of transcript per million mapped reads (FPKM). We used DESeq2^70^ to detect differential gene expression between the mutant and WT with criteria of a *P*-value ≤ 0.01 and |log2FoldChange| ≥3.00. We performed gene ontology (GO) function and pathway enrichment analyses using the GO seqR package^71^.

## Supplementary information

Supplementary Figure S1-S4

Supplementary Table S1

Supplementary Table S2

Supplementary Table S3

Supplementary Table S4

Supplementary Table S5

Supplementary Table S6

Supplementary Table S7

## Data Availability

All raw data, including resequencing and RNA-seq data, were submitted to the SRA (http://www.ncbi.nlm.nih.gov/sra/). In addition to genome sequences, protein sequences and CDSs can be found in the database (https://solgenomics.net).
