# Supplementary material for: OBV (obscure vein), a C2H2 zinc finger transcription factor, positively regulates chloroplast development and bundle sheath extension formation in tomato (Solanum lycopersicum) leaf veins
Source: Hortic Res. 2021 Nov 1;8:230. doi: 10.1038/s41438-021-00659-z (PMC8558323; doi:10.1038/s41438-021-00659-z)
Supplement: Supplementary file 1 — Supplementary Figure S1-S4 [file 41438_2021_659_MOESM1_ESM.docx]

**Supplementary data**

**Figure S1.** **Electron microscope observations of M82 and IL5-4-5-44.**

(a) SEM observations of M82 and IL5-4-5-44. (b) Analysis of the ratio of open stomata to total stomatal number in the lower epidermal cells. (c) TEM observations of M82 and IL5-4-5-44. (d) Analysis of chloroplast number in the vascular bundle. GC, guard cell; Chl, chloroplast; SG, starch granule; T, thylakoid. Scale bars: (a1–a4) 100 μm, (a5–a6) 200 μm, (c1–c2) 5 μm, (c3–c4) 0.5 μm, (c5–c6) 0.2 μm. Asterisks indicate statistically significant differences determined using a *t*-test (**P*<0.05; ***P*<0.01).

**Figure S2. Amino acid alignment and phylogenetic tree analysis of *OBV* from various species.**

(a) Phylogenetic analysis of *OBV*. Numbers shown at the tree forks indicate the frequency of occurrence among all bootstrap iterations performed. Scale bars, 0.05 cm. (**b)** Alignment of *OBV* sequences. Black and gray backgrounds indicate identical and similar amino acids, respectively. The locations of the four highly-conserved subdomains are indicated with black lines on top of the sequences. The red arrow represents the amino acid at position 135. Sl, *Solanum lycopersicum*; Sp, *Solanum* *pennellii*; Sc, *Solanum* *chilense*; St, *Solanum* *tuberosum*; Can, *Capsicum* *annuum*; Cb, *Capsicum* *baccatum*; Nt, *Nicotiana tabacum*; Cc, *Capsicum* *chinense*; Car, *Coffea* *arabica*; Gm, *Glycine* *max*; Vv, *Vitis* *vinifera*; Os, *Oryza* *sativa*.

**Figure S3. Electron microscope observations of OE-*OBV* and M82.**

(a) Expression levels of *OBV* in OE-*OBV* leaf veins compared with that of M82. (b) Chlorophyll content in the leaf veins of OE-*OBV* and M82. (c) SEM observations of OE-*OBV* and M82. (d) Analysis of the ratio of open stomatal to total stomatal number in the lower epidermal cells. (e) TEM observations of OE-*OBV* and M82. (f) Analysis of chloroplast number in the vascular bundle. GC, guard cell; Chl, chloroplast; SG, starch granule. Scale bars: (c1–c4) 100 μm, (c5–c6) 200 μm, (e1–e2) 5 μm, (e3–e4) 0.5 μm, (e5–e6) 0.2 μm. Asterisks indicate statistically significant differences determined using a *t*-test (**P*<0.05; ***P*<0.01).

**Figure S4. RNA-seq results of mesophyll and veins in Cris-*OBV* and WT plants.**

(a) Volcano plots of the DEGs between the mesophyll and veins in WT. (b) KEGG enrichment results of the DEGs between the mesophyll and veins in WT. (c) Volcano plots of the DEGs between the mesophyll and veins in Cris-*OBV*. (d) KEGG enrichment results of the DEGs between the mesophyll and veins in Cris-*OBV*. (a and c) Each point corresponds to a reference sequence gene. Red and blue represent upregulated and downregulated genes, respectively.
